# Characterization of plaque phenotypes exhibiting an elevated pericoronary adipose tissue attenuation: insights from the REASSURE-NIRS registry

**DOI:** 10.1007/s10554-023-02907-w

**Published:** 2023-06-29

**Authors:** Satoshi Kitahara, Yu Kataoka, Hiroyuki Miura, Tatsuya Nishii, Kunihiro Nishimura, Kota Murai, Takamasa Iwai, Hideo Matama, Satoshi Honda, Masashi Fujino, Shuichi Yoneda, Kensuke Takagi, Fumiyuki Otsuka, Yasuhide Asaumi, Yusuke Fujino, Kenichi Tsujita, Rishi Puri, Stephen J. Nicholls, Teruo Noguchi

**Affiliations:** 1 Department of Cardiology, Kashiwa Kousei General Hospital, 617 Shikoda, Kashiwa, Chiba, 277-0862 Japan; 2 Department of Advanced Cardiovascular Medicine, Graduate School of Medical Sciences, Kumamoto University, 1-1-1 Chuo-Ku, Honjo, Kumamoto, 860-8556 Japan; 3 Department of Cardiovascular Medicine, National Cerebral & Cardiovascular Center, 6-1 Kishibe-Shimmachi, Suita, Osaka, 564-8565 Japan; 4 Department of Radiology, National Cerebral & Cardiovascular Center, 6-1 Kishibe-Shimmachi, Suita, Osaka, 564-8565 Japan; 5 Department of Preventive Medicine and Epidemiology, National Cerebral & Cardiovascular Center, 6-1 Kishibe-Shimmachi, Suita, Osaka, 564-8565 Japan; 6 Department of Cardiovascular Medicine, Graduate School of Medical Sciences, Kumamoto University, 1-1-1 Honjo, Chuo-Ku, Kumamoto, 860-8556 Japan; 7 Department of Cardiovascular Medicine, Cleveland Clinic, 9500 Euclid Avenue, Cleveland, OH 44195 USA; 8 Victorian Heart Institute, Monash University, 631 Blackburn Rd, Clayton, VIC 3168 Australia

**Keywords:** Lipid-rich plaque, Large plaque burden, Spotty calcification, Positive remodeling, Anti-inflammatory agents

## Abstract

Inflammation has been considered to promote atheroma instability. Coronary computed tomography angiography (CCTA) visualizes pericoronary adipose tissue (PCAT) attenuation, which reflects coronary artery inflammation. While PCAT attenuation has been reported to predict future coronary events, plaque phenotypes exhibiting high PCAT attenuation remains to be fully elucidated. The current study aims to characterize coronary atheroma with a greater vascular inflammation. We retrospectively analyzed culprit lesions in 69 CAD patients receiving PCI from the REASSURE-NIRS registry (NCT04864171). Culprit lesions were evaluated by both CCTA and near-infrared spectroscopy/intravascular ultrasound (NIRS/IVUS) imaging prior to PCI. PCAT attenuation at proximal RCA (PCAT_RCA_) and NIRS/IVUS-derived plaque measures were compared in patients with PCAT_RCA_ attenuation ≥ and < -78.3 HU (median). Lesions with PCAT_RCA_ attenuation ≥ -78.3 HU exhibited a greater frequency of maxLCBI_4mm_ ≥ 400 (66% vs. 26%, p < 0.01), plaque burden ≥ 70% (94% vs. 74%, p = 0.02) and spotty calcification (49% vs. 6%, p < 0.01). Whereas positive remodeling (63% vs. 41%, p = 0.07) did not differ between two groups. On multivariable analysis, maxLCBI_4mm_ ≥ 400 (OR = 4.07; 95%CI 1.12–14.74, p = 0.03), plaque burden ≥ 70% (OR = 7.87; 95%CI 1.01–61.26, p = 0.04), and spotty calcification (OR = 14.33; 95%CI 2.37–86.73, p < 0.01) independently predicted high PCAT_RCA_ attenuation. Of note, while the presence of only one plaque feature did not necessarily elevate PCAT_RCA_ attenuation (p = 0.22), lesions harboring two or more features were significantly associated with higher PCAT_RCA_ attenuation. More vulnerable plaque phenotypes were observed in patients with high PCAT_RCA_ attenuation. Our findings suggest PCAT_RCA_ attenuation as the presence of profound disease substrate, which potentially benefits from anti-inflammatory agents.

**Supplementary Information:**

The online version contains supplementary material available at 10.1007/s10554-023-02907-w.

## Introduction

Inflammation has been considered as an important driver which promotes atherosclerosis [1]. Recently, coronary computed tomography angiography (CCTA) has been shown to enable visualization of pericoronary adipose tissue (PCAT) attenuation, which reflects the degree of coronary artery inflammation [2–4]. Several observational studies reported the association of PCAT attenuation with future coronary events [5–7]. Pathophysiologically, coronary artery inflammation is recognized to induce the influx of lipidic materials into vessel wall [8, 9], leading to the formation of unstable plaque associated with future coronary events. However, it remains to be fully elucidated phenotypic features of coronary plaque presenting PCAT attenuation.

Near-infrared spectroscopy (NIRS) imaging quantitatively evaluates the degree of lipidic plaque materials in *vivo* [10]. This modality provides an opportunity to evaluate the association between in vivo coronary artery inflammation and lipidic plaque materials. Therefore, the current study sought to determine culprit plaque features in patients with coronary artery disease (CAD) associated with high coronary inflammation by analyzing both PCAT and NIRS imaging.

## Methods

### Study population

We retrospectively analyzed 111 consecutive patients with CAD who underwent both clinically indicated CCTA and NIRS/intravascular ultrasound (IVUS) imaging prior to percutaneous coronary intervention (PCI) (1 Aug. 2015 – 31 Dec. 2021) from the REASSURE-NIRS registry (REvelation of PAthophySiological PhenotypeS of VUlneRable Lipid-Rich PlaquE on Near-InfraRed Spectroscopy) (NCT 04864171) (Supplementary Fig. 1). Of these, the following subjects were excluded; those imaged by inadequate CCTA imaging protocol (n = 17), patients with poor quality of NIRS/IVUS images (n = 1), a case with in-stent restenosis lesion (n = 1), and the interval between CCTA and NIRS/IVUS imaging > 4 months (n = 23). As a consequence, the remaining 69 patients with 69 de novo target lesions were included into the current study analysis. Target lesion was defined as the segment receiving PCI. The median time from CCTA to PCI was 38 days. The study protocol conforms to the ethical guidelines of the 1975 Declaration of Helsinki, and it was approved by the institutional ethics committee (research project number: M30-084-6).

### CCTA protocol

CCTA was performed by the second and third-generation dual-source CT (DSCT) scanners (SOMATOM Definition Flash and SOMATOM Force; Siemens Healthcare, Forchheim, Germany). Retrospective ECG-gated spiral scan with tube current modulation or prospective ECG triggered high-pitch spiral scan was selected depending on the heart rate. Further scan parameters in the second and third-generation DSCT were as follows: section collimation 2 × 64 × 0.6-mm and 2 × 96 × 0.6-mm, gantry rotation 0.275 s and 0.25 s, respectively. Automated tube current modulation (CARE Dose4D, Siemens) and automatic tube-voltage selection (CARE kV, Siemens) were used with 240–280 mAs as qualified reference tube-current time products and 120-kV as reference tube-voltage. The images were reconstructed using iterative reconstruction (SAFIRE or ADMIRE, Siemens) with 0.6-mm slice thickness and 0.3-mm increments with a medium convolution kernel (I31f or Bv40). The current CCTA imaging protocol was complied with the SCCT guidelines for performance of coronary computed tomographic angiography [11].

### PCAT analysis

Semi-automated software (Aquarius 3D Workstation, TeraRecon Inc., San Mateo, CA, USA) was used to measure PCAT attenuation at the proximal right coronary artery (PCAT_RCA_ attenuation) and at target lesion (PCAT_Lesion_ attenuation), respectively. Analysis of PCAT_RCA_ attenuation involved the proximal 10–50 mm of the RCA [2, 3, 12]. PCAT_Lesion_ attenuation was measured at target lesions with the proximal and distal borders of the analysis region which were defined as the proximal and distal ends of the target lesion [2, 13, 14]. A centerline was applied to both the proximal segment of RCA and the target lesion, and then adventitia was demarcated from surrounding adipose tissue. Adipose tissue was defined as all voxels between − 190 Hounsfield units (HU) and − 30 HU within a predefined volume of interest. This volume interest included three-dimensional concentric layers extending outward from the operator traced vessel wall to an extent of the reference diameter of the target lesion. PCAT attenuation was automatically quantified as the mean attenuation of all voxels within by the manually defined centerline and vessel wall. PCAT measurement was conducted by two independent experienced operators who were blinded to clinical data (SK, HM).

### NIRS/IVUS imaging

NIRS/IVUS imaging was conducted prior to PCI to evaluate the entire target vessel. After intracoronary administration of nitroglycerin (100–300 μg), the imaging catheter (TVC Insight^™^ or Dualpro^™^, Infraredx, Bedford, MA, USA) was automatically pullbacked from the most distal site of the target artery at a speed of 0.5 mm/sec and 960 rpm (TVC Insight^™^) or 2.0 mm/sec and 1800 rpm (Dualpro^™^) [15]. Makoto^®^ system (Infraredx, Bedford, MA, USA) was used to analyze obtained chemogram data [16]. MaxLCBI_4mm_ at target lesion was used for the analysis [17]. Qualitative analysis on IVUS imaging was performed to evaluate (1) plaque area, (2) remodeling index (RI), and (3) spotty calcification. Plaque area was defined as the difference in area occupied by the lumen and external elastic membrane (EEM) borders. Total atheroma volume was calculated by the summation of the plaque area in each measured image. Plaque burden was calculated as the percentage of total atheroma volume to EEM. RI was assessed in short axis view of the vessel by the following formula;

RI = (cross-sectional EEM area at minimum lumen area) / (EEM area of the proximal reference segment).

RI ≥ 1.05 was considered as positive remodeling[18]. The presence of calcification was assessed at every 1-mm cross-sectional image, and then the arc of calcification was measured. Spotty calcification was defined as the presence of lesions 1 to 4 mm in length containing an arc of calcification < 90°[19]. NIRS/IVUS images were analyzed by physicians who were blinded to the clinical characteristics of the patients (KM and YK).

### Quantitative coronary angiography analysis

Quantitative coronary angiography (QCA) analysis was performed at target and non-target lesions by using off-line commercially available software (QAngio® XA, Medis, Leiden, the Netherlands). QCA analysis included minimal lumen diameter, percent diameter stenosis, lesion length and reference vessel diameter.

### Statistical analysis

Continuous variables were expressed as the mean ± standard deviation and compared using the t test if data were normally distributed. Non-normally distributed continuous data were summarized as the median (interquartile range) and compared using the Wilcoxon rank sum test. Categorical variables were compared using the Fisher exact test or the Chi-square test as appropriate. Spearman’s rank-order correlation was used to examine the relationship of PCAT_RCA_ attenuation with maxLCBI_4mm_. Nominal logistic regression analysis was conducted to determine NIRS/IVUS plaque features associated with high PCAT_RCA_ attenuation. Parameters with P value < 0.10 in univariable analysis were entered into multivariable one. Receiver-operating characteristic (ROC) curve analyses were conducted to evaluate the ability of clinical features (age, sex, dyslipidemia and LDL-C ≥ 1.8 mmol/L) and PCAT_RCA_ for the prediction of NIRS/IVUS-derived vulnerable plaque [20, 21]. According to the published paper analyzing lipid (n = 40) and non-lipid (n = 15) plaques [22], the expected difference in the frequency of low attenuation plaque is considered as 10% between these two types of plaques. A sample of 56 lesions will be required for 90% power at a two-sided alpha level of 0.05 to detect a nominal difference of 10%, assuming a standard deviation of 10%. All P values < 0.05 were considered statistically significant. All analyses were performed with JMP version 14 (SAS Institute, Cary, NC).

## Results

### Clinical demographics of study subjects

The median PCAT_RCA_ attenuation was -78.3 HU. Study subjects were stratified into two groups according to the median PCAT_RCA_ attenuation. Baseline clinical characteristics of study population are summarized in Table 1. Patients were predominantly male (80%) with a high prevalence of hypertension (72%), dyslipidemia (86%) and type 2 diabetes mellitus (33%). Eighty-six percent of study subjects presented stable CAD. With regard to the use of anti-atherosclerotic medical therapies, 59% and 14% of study subjects received statin and ezetimibe, respectively. Lipids measures and c-reactive protein levels did not differ between two groups. (Table 1).

**Table 1:** Clinical Characteristics

	Total (n = 69)	PCAT_RCA_ attenuation ≥ -78.3HU (n = 35)	PCAT_RCA_ attenuation < -78.3HU (n = 34)	p value
Age, years	71 (55–77)	64 (52–77)	71 (61–76)	0.31
Male, n (%)	55 (80%)	28 (80%)	27 (79%)	0.95
BMI (kg/m^2^)	23.9 (21.9–26.7)	24.1 (20.8–27.2)	23.9 (22.0–25.9)	0.74
Hypertension, n (%)	50 (72%)	24 (69%)	26 (76%)	0.46
Dyslipidemia, n (%)	59 (86%)	29 (83%)	30 (88%)	0.53
T2DM, n (%)	23 (33%)	12 (34%)	11 (32%)	0.86
Current Smoking, n (%)	12 (17%)	5 (14%)	7 (21%)	0.49
Prior PCI, n (%)	25 (36%)	13 (37%)	12 (35%)	0.87
Diagnosis of CAD				
Stable CAD, n (%)	59 (86%)	31 (89%)	28 (82%)	0.46
ACS, n (%)	10 (14%)	4 (11%)	6 (18%)	
Medication use				
Aspirin, n (%)	19 (28%)	13 (37%)	6 (18%)	0.07
P2Y12 inhibitor, n (%)	10 (14%)	8 (23%)	2 (6%)	0.05
Statin, n (%)	41 (59%)	21 (60%)	20 (59%)	0.92
Ezetimibe, n (%)	10 (14%)	4 (11%)	6 (18%)	0.46
β-blocker, n (%)	26 (38%)	15 (43%)	11 (32%)	0.37
ACE-I / ARB, n (%)	29 (42%)	9 (26%)	20 (59%)	**0.01**
Laboratory data				
eGFR (mL/min/1.73m^2^)	66 (60–73)	69 (61–83)	63 (59–70)	0.06
LDL-C (mmol/l)	2.59 (2.09–3.41)	2.56 (2.07–3.28)	2.61 (1.93–3.41)	0.65
HDL-C (mmol/l)	1.24 (1.06–1.42)	1.29 (1.14–1.42)	1.11 (1.03–1.47)	0.42
Triglyceride (mmol/l)	1.29 (0.90–1.98)	1.06 (0.88–1.69)	1.46 (0.95–2.13)	0.11
HbA1c (%)	5.8 (5.6–6.5)	5.9 (5.6–6.4)	5.8 (5.6–6.4)	0.83
CRP (μg/l)	900 (300–2100)	500 (200–1800)	1100 (500–2200)	0.50

Non-normally distributed continuous data were summarized as the median (interquartile range)

*ACE-I=* angiotensin converting enzyme inhibitor, *ACS=* acute coronary syndrome, *ARB=* angiotensin ll receptor blocker, *BMI=* body mass index, *CAD=* coronary artery disease, *eGFR=* estimated glomerular filtration rate, *HDL=* high density lipoprotein, *LAD=* left anterior descending artery, *LDL=* low density lipoprotein, *PCAT=* pericoronary adipose tissue, *PCI=* percutaneous coronary intervention, *RCA=* right coronary artery, *T2DM=* Type2 diabetes mellitus

### Coronary angiographic features of analyzed lesions

Table 2 shows characteristics of 69 analyzed lesions. Over two-thirds of analyzed lesions were located within the left anterior descending artery. There were no significant differences in QCA measures between two groups.

**Table 2:** Angiographical and imaging analysis

	Total (n = 69)	PCAT_RCA_ attenuation ≥ -78.3HU (n = 35)	PCAT_RCA_ attenuation < -78.3HU (n = 34)	P value
Culprit lesion				
LMT, n (%)	4 (6%)	3 (9%)	1 (3%)	
LAD, n (%)	46 (67%)	22 (63%)	24 (71%)	
LCX, n (%)	6 (9%)	3 (9%)	3(9%)	
RCA, n (%)	13 (19%)	7 (20%)	6 (18%)	
Proximal segment, n (%)	41 (59%)	23 (66%)	18 (53%)	0.28
QCA analysis				
%Diameter stenosis (%)	68.0 ± 16.5	67.5 ± 17.2	68.5 ± 15.9	0.56
Lesion length (mm)	19.0 (13.6–22.9)	17.8 (12.9–21.2)	19.8 (14.2–23.8)	0.81
Reference diameter (mm)	3.5 ± 0.7	3.6 ± 0.6	3.5 ± 0.8	0.47
PCAT analysis				
PCAT_RCA_ attenuation (HU)	− 78.3 (− -86.3 to − 71.7)	− 71.9 (− 76.0 to − 69.2)	− 86.3 (− 91.3 to − 82.1)	< 0.01
PCAT_Lesion_ attenuation (HU)	− 79.3 ± 12.2	− 72.4 ± 9.4	− 86.5 ± 10.6	< 0.01
NIRS measurement				
MaxLCBI_4mm_	403 ± 219	466 ± 210	338 ± 211	**0.01**
MaxLCBI_4mm_ ≥ 400, n (%)	32 (46%)	23 (66%)	9 (26%)	** < 0.01**
IVUS measurement				
Plaque burden (%)	59 (54–64)	61 (58–65)	56 (47–62)	** < 0.01**
Plaque burden ≥ 70%, n (%)	58 (84%)	33 (94%)	25 (74%)	**0.02**
Remodeling index	1.07 (0.97–1.28)	1.15 (0.98–1.29)	1.02 (0.97–1.17)	0.10
Remodeling index ≥ 1.05, n (%)	36 (52%)	22 (63%)	14 (41%)	0.07
Spotty calcification, n (%)	19 (28%)	17 (49%)	2 (6%)	** < 0.01**

Continuous data are represented as means ± standard deviation if data were normally distributed Non-normally distributed continuous data were summarized as the median (interquartile range)

*IVUS=* intravascular ultrasound, *LAD=* left anterior descending artery, *LCX=* left circumflex artery, *LMT=* left main trunk, *MaxLCBI*_4mm_= maximum 4 mm Lipid Core Burden Index, *NIRS* = near-infrared spectroscopy, *PCAT=* pericoronary adipose tissue, *RCA=* right coronary artery, *QCA=* quantitative coronary angiography

Significant P value <0.05 is indicated in bold

### Characteristics of PCAT and NIRS/IVUS measures

On PCAT analysis, lesions with PCAT_RCA_ attenuation ≥ -78.3 HU exhibited a higher PCAT_Lesion_ attenuation. NIRS/IVUS imaging analysis demonstrated a greater maxLCBI_4mm_ (466 ± 210 vs. 338 ± 211, p = 0.01) at lesions with PCAT_RCA_ attenuation ≥ -78.3 HU, accompanied by a higher frequency of maxLCBI_4mm_ ≥ 400 (66% vs. 26%, p < 0.01) (Table 2). In addition, a greater plaque burden, a higher proportion of plaque burden ≥ 70% and spotty calcification were observed at lesions with PCAT_RCA_ attenuation ≥ -78.3 HU. There was a trend toward a greater remodeling index and a higher frequency of positive remodeling in those with PCAT_RCA_ attenuation ≥ -78.3 HU, but these comparisons did not meet statistical significance (Table 2).

**Fig. 1:**
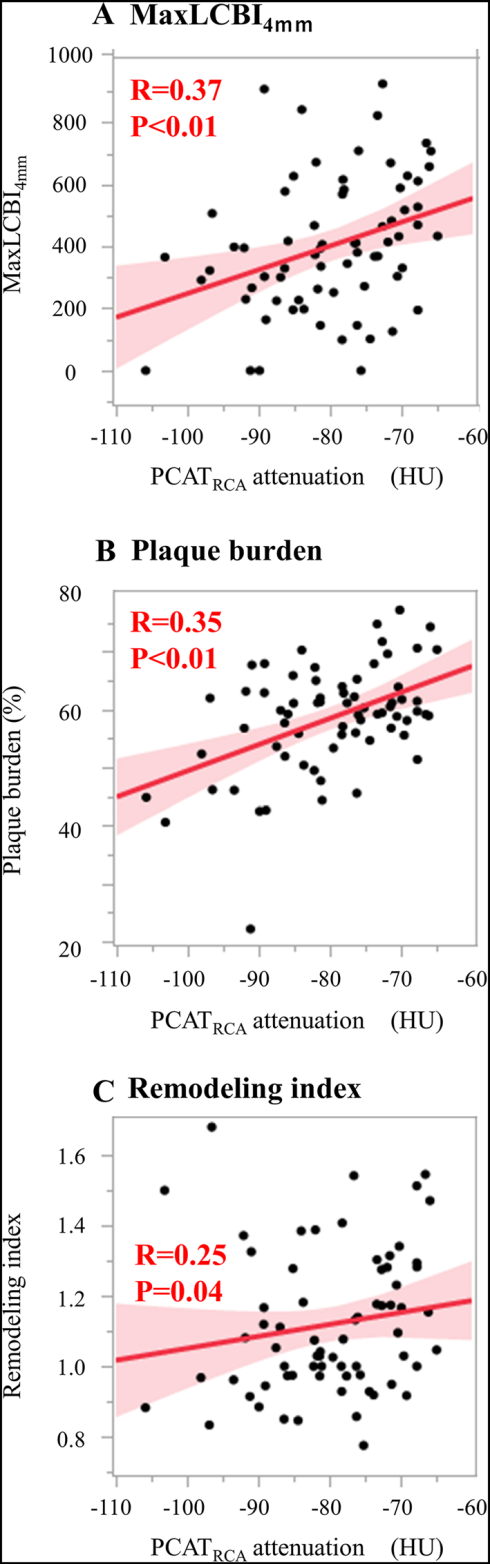
The correlation between NIRS/IVUS-derived plaque features and PCAT_RCA_ attenuation. **A** MaxLCBI_4mm_, **B** Plaque burden, **C** Remodeling index. *IVUS=* ntravascular ultrasound, *NIRS=* near infrared spectroscopy, *PCAT=*  pericoronary adipose tissue, *maxLCBI*_4mm_ = maximum-4 mm lipid core burden index, *RCA=* right coronary artery

Figure 1 illustrated the relationships of PCAT_RCA_ attenuation with maxLCBI_4mm_, plaque burden and remodeling index, respectively. MaxLCBI_4mm_ (R = 0.37, p < 0.01, Fig. 1A), plaque burden (R = 0.35, p < 0.01, Fig. 1B) and remodeling index (R = 0.25, p = 0.04, Fig. 1C) were positively associated with PCAT_RCA_ attenuation.

With regards to PCAT_Lesion_, PCAT_Lesion_ attenuation were significantly correlated with PCAT_RCA_ attenuation (R = 0.70, p < 0.01) **(**Supplementary Fig. 2) and plaque burden (R = 0.29, 0 = 0.01), respectively. However, the relationships of PCAT_Lesion_ attenuation with maxLCBI_4mm_ (R = 0.23, p = 0.05) and remodeling index (R = 0.16, p = 0.20) were not statistically significant (Supplementary Fig. 3 a-c). Furthermore, PCAT_Lesion_ attenuation did not differ between lesions with and without spotty calcification (Supplementary Fig. 4).

### Factors associated with high PCAT_RCA_ attenuation

Uni- and multivariable logistic regression analyses were conducted to identify an independent NIRS/IVUS-derived feature associated with PCAT_RCA_ attenuation ≥ -78.3 HU. Univariable analysis revealed that maxLCBI_4mm_ ≥ 400, maximum plaque burden ≥ 70% and the presence of spotty calcification were associated with high PCAT_RCA_ attenuation (Table 3). On multivariable analysis, these plaque measures emerged as the independent predictors of PCAT_RCA_ attenuation ≥ -− 8.3 HU (maxLCBI_4mm_ ≥ 400: OR = 4.07, 95%CI 1.12–14.74, p = 0.03, maximal plaque burden ≥ 70%: OR = 7.87, 95%CI 1.01–61.26, p = 0.04, spotty calcification: OR = 14.33, 95%CI 2.37–86.73, p < 0.01, Table 3) but not positive remodeling (OR = 1.26, 95%CI 0.36–4.45, p = 0.72).

**Table 3:** Uni- and multivariable analysis for detecting high PCAT_RCA_ attenuation

	Univariable analysis			Multivariable analysis*		
	OR	95%CI	P value	OR	95%CI	P value
Age	0.97	0.93–1.01	0.15	0.99	0.94–1.05	0.72
Male	1.04	0.32–3.35	0.95	1.05	0.23–4.71	0.95
Hypertension	0.67	0.23–1.95	0.46			
Dyslipidemia	0.64	0.16–2.52	0.53			
Diabetes mellitus	1.09	0.40–2.97	9.86			
Statin use	1.05	0.40–2.74	0.92			
LDL-C	1.00	0.99–1.01	1.00			
MaxLCBI_4mm_ ≥ 400	5.32	1.89–14.96	** < 0.01**	4.07	1.12–14.74	**0.03**
Maximal plaque burden ≥ 70%	5.94	1.18–29.95	**0.03**	7.87	1.01–61.26	**0.049**
Positive remodeling†	2.42	0.92–6.36	0.07	1.26	0.36–4.45	0.72
Spotty calcification	15.11	3.13–72.99	** < 0.01**	14.33	2.37–86.73	** < 0.01**

*CI=* Confidence interval, *LDL=* low density lipoprotein, *MaxLCBI*_*4mm*_= maximum-4 mm Lipid Core Burden Index, *OR=* Odds ratio, *PCAT=* pericoronary adipose tissue, *RCA=* right coronary artery

†Positive remodeling was defined as remodeling index ≥ 1.05

*Multivariable logistic regression analysis with factors that were significant in the univariable analysis (p value < 0.10), age, and male

Significant P value <0.05 is indicated in bold

**Fig. 2:**
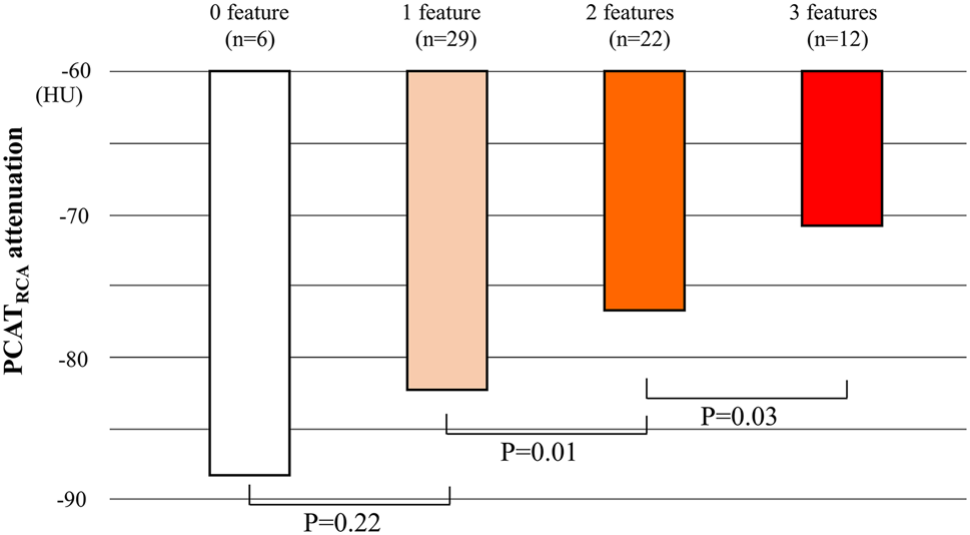
The median PCAT_RCA_ attenuation and the number of NIRS/IVUS-derived plaque features. *IVUS=* intravascular ultrasound, *NIRS=*  near infrared spectroscopy, *PCAT=* pericoronary adipose tissue, *RCA=*  right coronary artery

**Fig. 3:**
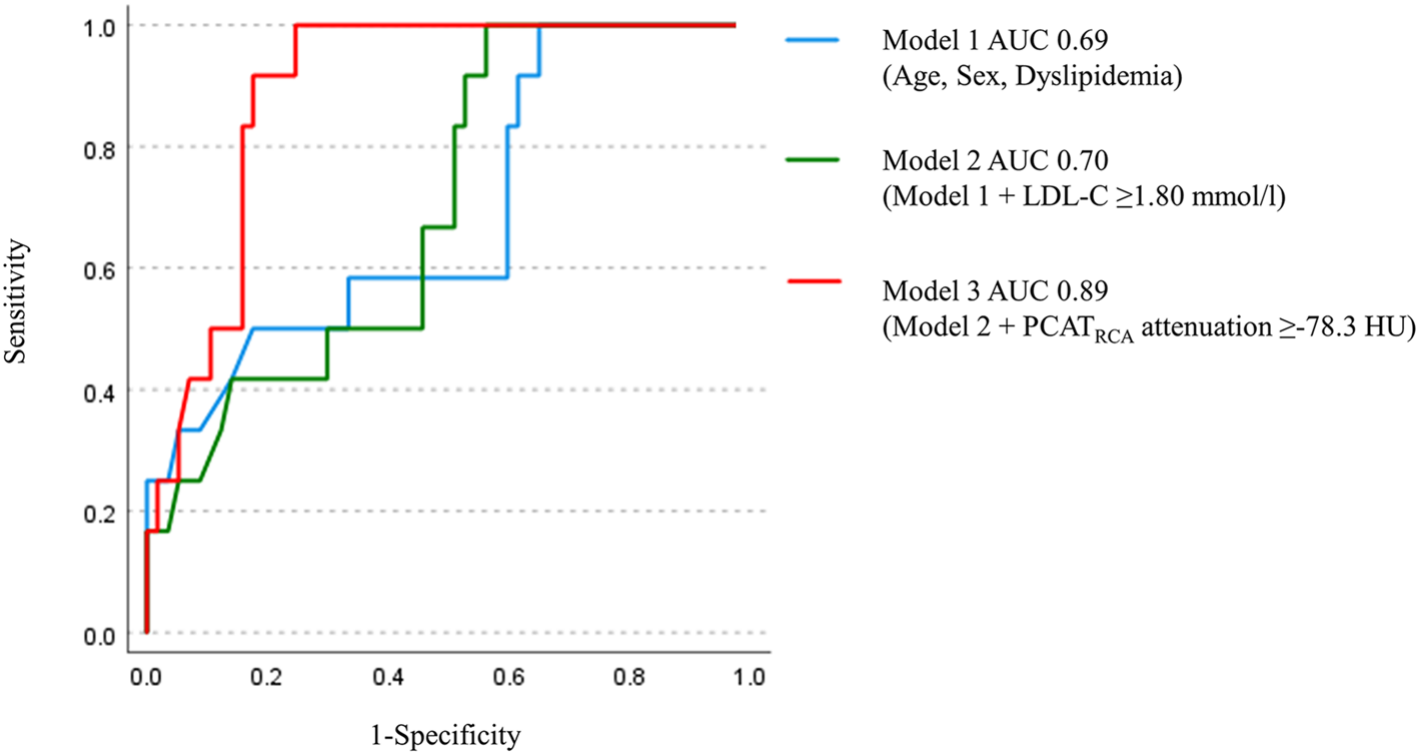
Receiver operating characteristics curve analysis to predict vulnerable plaques. Three models were used to evaluate the predictive ability of NIRS/IVUS-derived vulnerable plaque (= maxLCBI_4mm_ > 400 + plaque burden > 70% + spotty calcification). Model 1: age, sex, and dyslipidemia (light blue). Model 2: model 1+LDL-C ≥ 1.8 mmol/L) (green). Model 3: model 2+PCAT_RCA_ attenuation ≥ -78.3 HU (red)

**Fig. 4:**
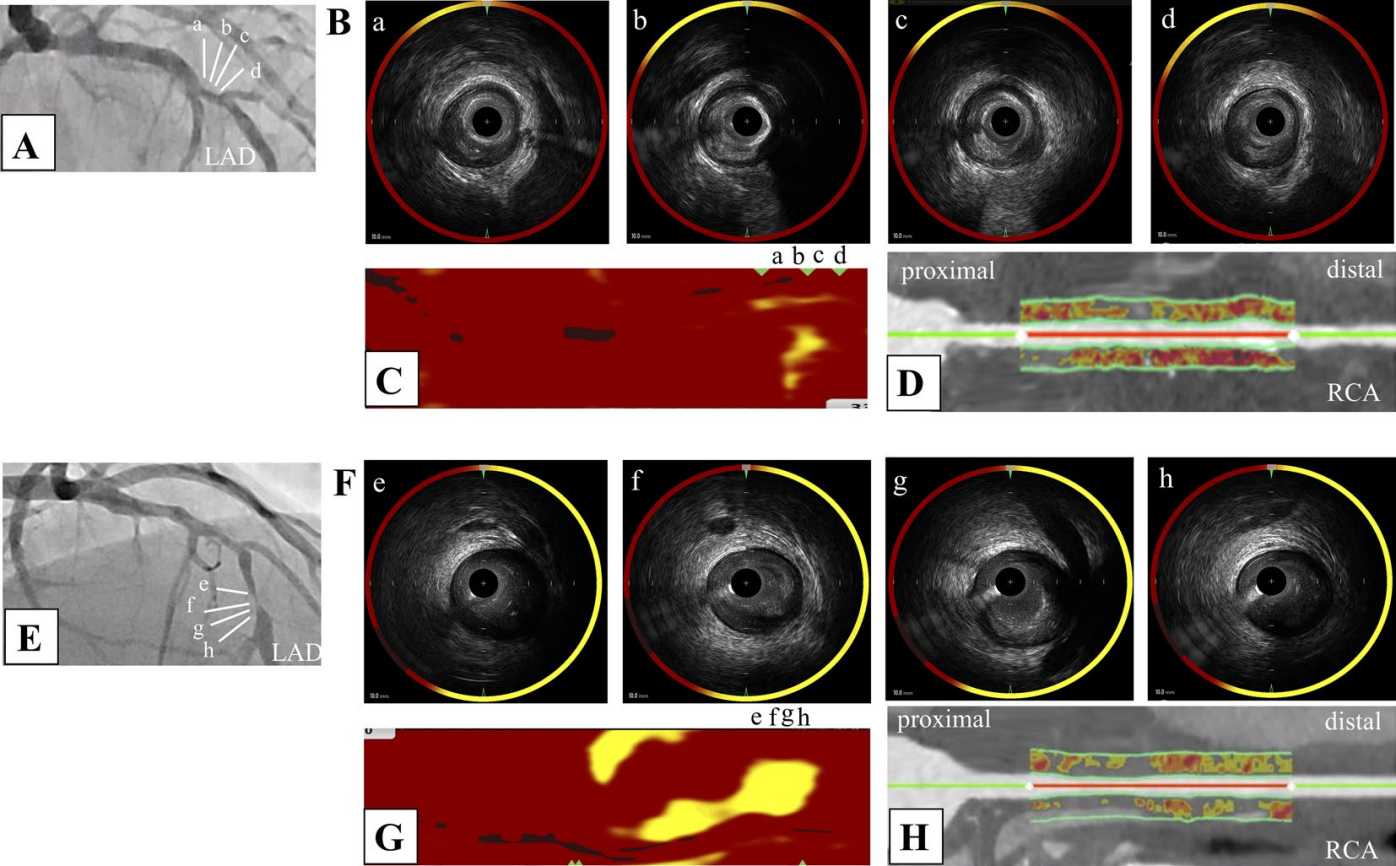
Representative cases with or without high vascular inflammation. **A** 77 years old male with stable coronary artery disease. Coronary angiography demonstrated that moderate stenosis in his middle left descending artery (LAD). **B** Grey-scale intravascular ultrasound (IVUS) showed calcified plaque with no attenuation in the culprit lesion. **C** Near-infrared spectroscopy chemogram revealed that there was less lipid accumulation in the culprit lesion (maxLCBI_4mm_ = 292). **D** In coronary computed tomography angiography (CCTA) analysis, his pericoronary adipose tissue attenuation at proximal RCA was low (PCAT_RCA_ attenuation = − 98.1 HU). **E** 85 years old male with stable coronary artery disease. Coronary angiography demonstrated that severe stenosis in his middle LAD. **F** Grey-scale IVUS showed low echoic plaque with attenuation in the culprit lesion. **G** NIRS chemogram revealed that there was large lipid burden in the culprit lesion (maxLCBI_4mm_ = 465). **H** In CCTA analysis, he had high coronary artery inflammation (PCAT_RCA_ attenuation = − 72.7 HU)

PCAT_RCA_ attenuation was compared in lesions stratified according to the number of NIRS/IVUS-derived features (Fig. 2). PCAT_RCA_ attenuation at lesions with one NIRS/IVUS-derived plaque feature was similar to that at lesions without any plaque features (p = 0.22). By contrast, PCAT_RCA_ attenuation was greater in association with an increasing number of plaque features (Fig. 2). ROC curve analyses were conducted to evaluate the ability of PCAT_RCA_ for the prediction of NIRS/IVUS-derived vulnerable plaque (Fig. 3). PCAT_RCA_ exhibited an excellent predictive ability of vulnerable plaque [area under the curve (AUC) = 0.82, p < 0.01]. Furthermore, the addition of PCAT_RCA_ with clinical features (age, sex, dyslipidemia and LDL-C ≥ 1.8 mmol/L) improved its prediction [model 1 (age, sex and dyslipidemia): AUC = 0.69, model 2 (age, sex, dyslipidemia and LDL-C ≥ 1.8 mmol/L): AUC = 0.70, model 3 (age, sex, dyslipidemia, LDL-C ≥ 1.8 mmol/L and PCAT_RCA_ attenuation ≥ -78.3 HU): AUC = 0.89] (Fig. 3). Two representative cases are shown in Fig. 4.

## Discussion

While evaluation of coronary artery inflammation has a potential to stratify future risks of coronary events [1], whether this approach truly reflects disease substrate associated with the occurrence of coronary events remains to be fully determined yet. In the current study, patients with a higher CCTA-derived PCAT_RCA_ attenuation exhibited a greater atheroma burden at target lesions. Furthermore, accumulation of lipidic plaque component and spotty calcification were more frequently observed in patients with a higher PCAT_RCA_ attenuation. These findings highlight the ability of PCAT_RCA_ attenuation to non-invasively identify high-risk plaque phenotypes causing future coronary events.

Pericoronary adipose tissue has been recently recognized as an important contributor to atherosclerosis via secreting proinflammatory adipokines and cytokines. Pathophysiologically, proinflammatory stimuli secreted from pericoronary adipose tissue diffusely propagate into the adventitia, media, and intima. These properties interact with endothelial cells and vascular smooth muscle cells, which cause the formation, progression and destabilization of atherosclerotic plaques [23, 24]. In particular, inflammatory activities could promote the influx of lipidic materials within vessel wall [8, 9]. In the current study, in addition to plaque area, maxLCBI_4mm_ at target lesions increased in association with PCAT_RCA_ attenuation in vivo. One recent study characterized coronary atherosclerosis as the concomitance of macrophage polarization with a greater expression of interleukin-6, tumor necrosis factor-alpha and monocyte chemotactic protein-1 [25]. Our findings as well as the aforementioned study suggest in vivo relationship of inflammatory activity at pericoronary adipose tissue with the formation of lipidic coronary atheroma in patients with CAD.

Spotty calcification is a phenotypic feature of coronary atheroma which reflects active form of disease stimulated by inflammation. Mechanistically, inflammatory cytokines such as tumor necrosis factor-alpha activate osteogenic differentiation and mineralization of vascular cells, which contribute to early stages of plaque calcification [26, 27]. The current analysis observed an increased frequency of spotty calcification in patients with high PCAT_RCA_ attenuation. This finding suggests that patients with vascular inflammation may exhibit a greater degree of plaque inflammation, which could promote its instability and then cause future coronary events. Intravascular imaging studies showed an accelerated progression and more unstable features at spotty calcified lesions [28]. These observations support evaluation of PCAT_RCA_ attenuation as a tool to measure plaque inflammation associated with its progression/rupture.

The current study underscores a clustering of phenotypic plaque features in patients exhibiting an elevated vascular inflammation. As shown in Fig. 2, PCAT_RCA_ attenuation did not increase even if one plaque feature existed, whereas clustering of more than two features associates with a higher level of PCAT_RCA_ attenuation. The cardiovascular effects of clustering plaque features have been reported by the PROSPECT study [29]. In this study, cardiovascular events’ risks incrementally elevate in association with the number of plaque features including its volume and thin-cap fibroatheroma. Another study elucidated substantially progressive disease substrate in patients with multiple vulnerable plaque characteristics [30]. These observations provide mechanistic insights into the potential effect of vascular inflammation to induce the accumulation of multiple atherosclerotic features, which account for the predictive ability of PCAT_RCA_ attenuation to predict future risk of cardiovascular events.

The current study used PCAT_RCA_ attenuation as a marker reflecting inflammation of the entire coronary tree, whereas PCAT_Lesion_ may be better to evaluate lesion-related inflammation activity. In our analysis, while PCAT_Lesion_ was attenuation positively correlated with PCAT_RCA_ attenuation (R = 0.70, p < 0.01), the correlation of PCAT_Lesion_ attenuation with NIRS/IVUS-derived plaque features were weak. Most of published studies have measured PCAT attenuation at the proximal 40 mm segment of RCA, and they reported that PCAT_RCA_ predicted a future cardiovascular event [7]. This measurement of PCAT is based on pathophysiological and anatomical features of RCA. Adipose tissues abundantly exist at the proximal segment of RCA, which could better reflect inflammatory activity [2]. In addition, the number of side branches at this segment is low, and luminal diameter does not dynamically change throughout the proximal segment of RCA [3]. These anatomical characteristics are suitable to clearly visualize PCAT attenuation. In our study, maxLCBI_4mm_ was associated with PCAT_RCA_ but not PCAT_Lesion_ attenuation. Our observation also supports the measurement of PCAT attenuation at the proximal segment of RCA to more appropriately evaluate coronary artery inflammation.

To date, clinically adequate cut-off value of PCAT_RCA_ attenuation is not fully established yet. Tzolos et al. reported that PCAT_RCA_ attenuation ≥ -70.5 HU predicted future outcome[6], whereas other study showed its value ≥ -76.3 HU as a contributor for outcomes[7]. In the current study, we used the median value of PCAT_RCA_ attenuation in the entire subjects (-78.3 HU) as a cut-off value of PCAT_RCA_ attenuation. Further studies are required to identify the best cut-off value of PCAT_RCA_ attenuation associated with plaque instability and cardiovascular outcomes.

Considerable interests have recently focused on inflammation as a therapeutic target because of its biological properties. The CANTOS (Canakinumab Antiinflammatory Thrombosis Outcome Study) study demonstrated the clinical benefit of modulating interleukin-1β to reduce future atherosclerotic cardiovascular disease in patients with an elevated level of high-sensitivity c-reactive protein [31]. A significant reduction of cardiovascular events in patients with recent myocardial infarction receiving colchicine has been reported by COLCOT (Colchicine Cardiovascular Outcomes Trial) study [32]. These evidence indicates that patients with elevated inflammatory activity may benefit from anti-inflammatory agents. Given that PCAT imaging is a non-invasive modality to evaluate inflammation activity, this approach may be clinically applicable to identify those who require anti-inflammatory therapies. Future dedicated studies will be warranted to elucidate whether PCAT-guided anti-inflammatory therapy could improve cardiovascular outcomes.

Several caveats should be noted. Firstly, this study is a single-center retrospective observational study. Secondly, the sample size of the current study is relatively small, which may lead to the wide 95% CI in the multivariable analysis However, our calculation of study sample size shows that 56 lesions provide 90% power at a two-sided alpha level of 0.05 to detect a nominal difference of 10% (standard deviation = 10%). Thirdly, CCTA imaging was used according to each physician’s discretion. These may cause a potential bias to select study population. Fourthly, the use of lipid-lowering therapy and its intensity were decided by each physician. Fifthly, while one recent study showed the association of PCAT_RCA_ attenuation with monocyte chemoattractant protein-1 and interleukin-7 [31], the current study did not measure inflammatory biomarkers.

In conclusion, patients with an elevated PCAT_RCA_ attenuation more likely exhibited greater atheroma burden containing lipidic materials and spotty calcification. Of note, a higher PCAT_RCA_ attenuation was observed in association with an increased number of these phenotypic features. Our findings suggest that CCTA-derived PCAT_RCA_ attenuation has a potential to non-invasively identify patients harboring high-risk plaque phenotype who may require preventive medical therapies including anti-inflammation agent.

### Supplementary Information

Below is the link to the electronic supplementary material.

Supplementary file1 (TIF 767 kb)—Patient’s disposition. *CAD=* coronary artery disease, *CCTA=* coronary computed tomography angiography, *HU=* Hounsfield units, *IVUS=* intravascular ultrasound, *NIRS=* near infrared spectroscopy, *PCAT=* pericoronary adipose tissue, *PCI=* percutaneous coronary intervention, *RCA=* right coronary artery

Supplementary file2 (TIF 1080 kb)—The correlation between PCAT_Lesion_ attenuation and PCAT_RCA_ attenuation. *PCAT=* pericoronary adipose tissue. *RCA=* right coronary artery

Supplementary file3 (TIF 1887 kb)—The correlation between plaque features derived from NIRS/IVUS and PCAT_Lesion_ attenuation. **a** MaxLCBI_4mm_, **b** Plaque burden, **c** Remodeling index. *IVUS=* intravascular ultrasound, *NIRS= *near infrared spectroscopy, *PCAT=* pericoronary adipose tissue

Supplementary file4 (TIF 487 kb)—The relationships of PCAT_Lesion_ attenuation with spotty calcification. *PCAT=* pericoronary adipose tissue

## Abbreviations

AUC: Area under the curve
CAD: Coronary artery disease
CCTA: Coronary computed tomography angiography
DSCT: Dual-source CT
EEM: External elastic membrane
IVUS: Intravascular ultrasound
NIRS: Near-infrared spectroscopy
PCAT: Pericoronary adipose tissue
QCA: Quantitative coronary angiography
RCA: Right coronary artery
RI: Remodeling index
ROC: Receiver-operating characteristics
