# Supplementary figures and images for: Characterization of plaque phenotypes exhibiting an elevated pericoronary adipose tissue attenuation: insights from the REASSURE-NIRS registry
Source: Int J Cardiovasc Imaging. 2023 Jun 29;39(10):1943–52. doi: 10.1007/s10554-023-02907-w (PMC10589176; doi:10.1007/s10554-023-02907-w)

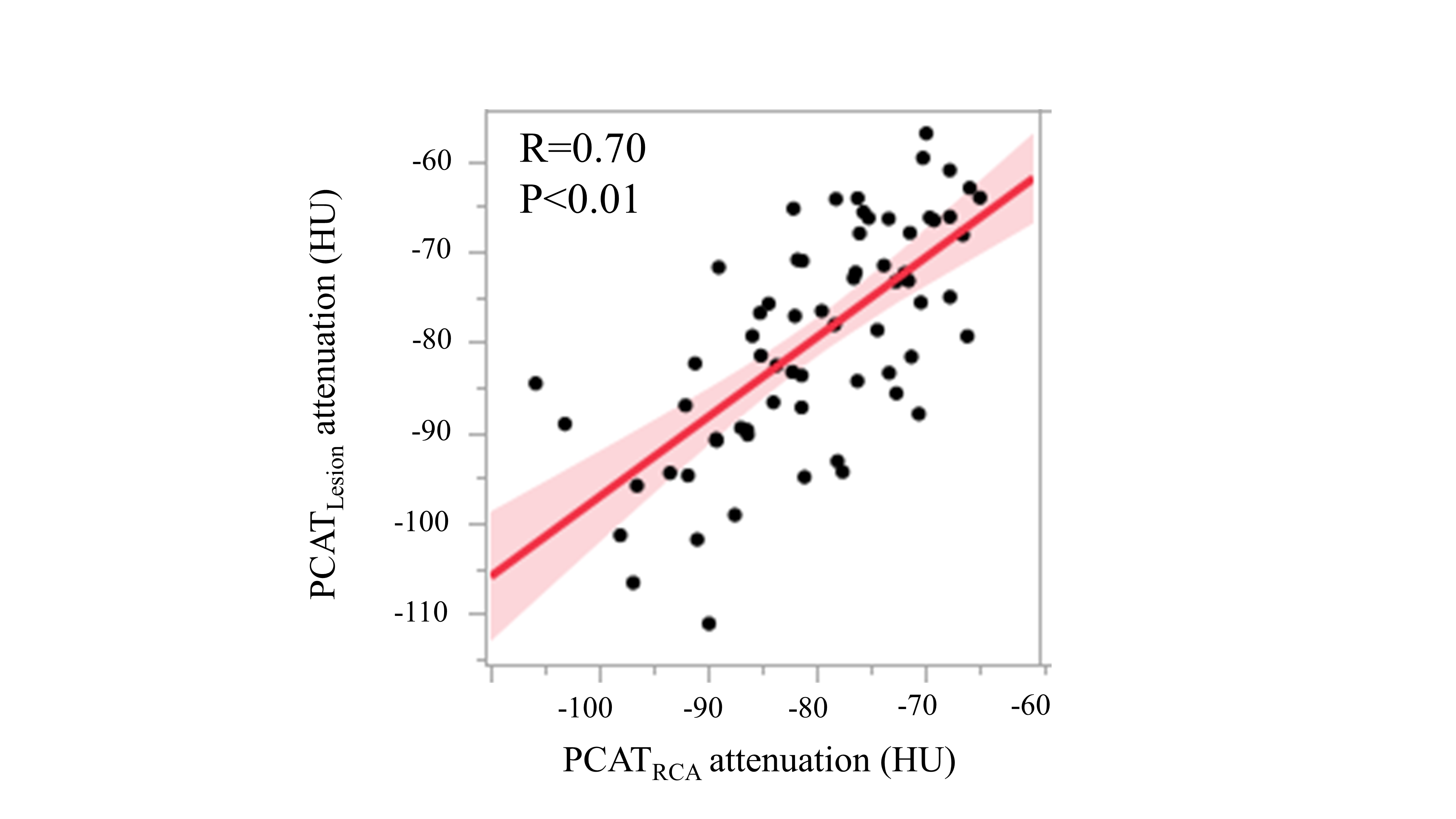

Supplement: Supplementary file 1 — Supplementary file1 (TIF 767 kb)—Patient’s disposition. CAD= coronary artery disease, CCTA= coronary computed tomography angiography, HU= Hounsfield units, IVUS= intravascular ultrasound, NIRS= near infrared spectroscopy, PCAT= pericoronary adipose tissue, PCI= percutaneous coronary intervention, RCA= right coronary artery [file 10554_2023_2907_MOESM1_ESM.tif]

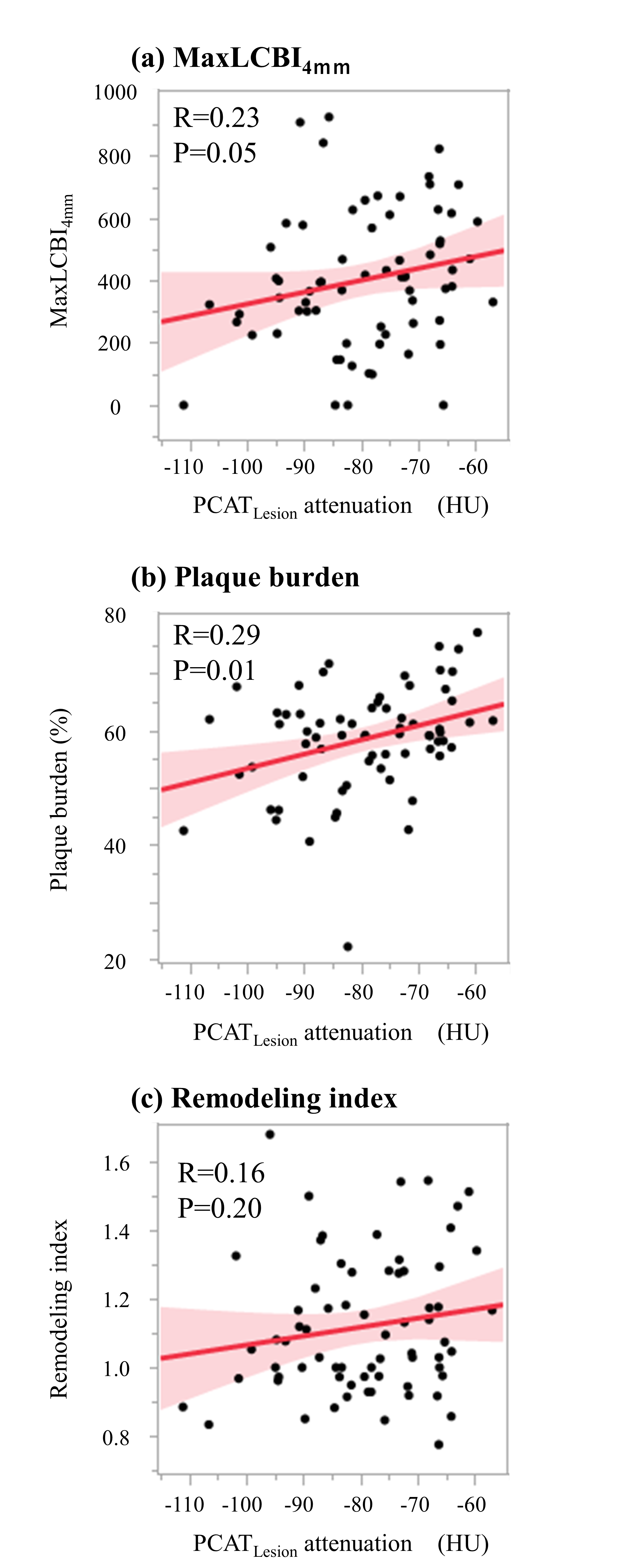

Supplement: Supplementary file 2 — Supplementary file2 (TIF 1080 kb)—The correlation between PCATLesion attenuation and PCATRCA attenuation. PCAT= pericoronary adipose tissue. RCA= right coronary artery [file 10554_2023_2907_MOESM2_ESM.tif]

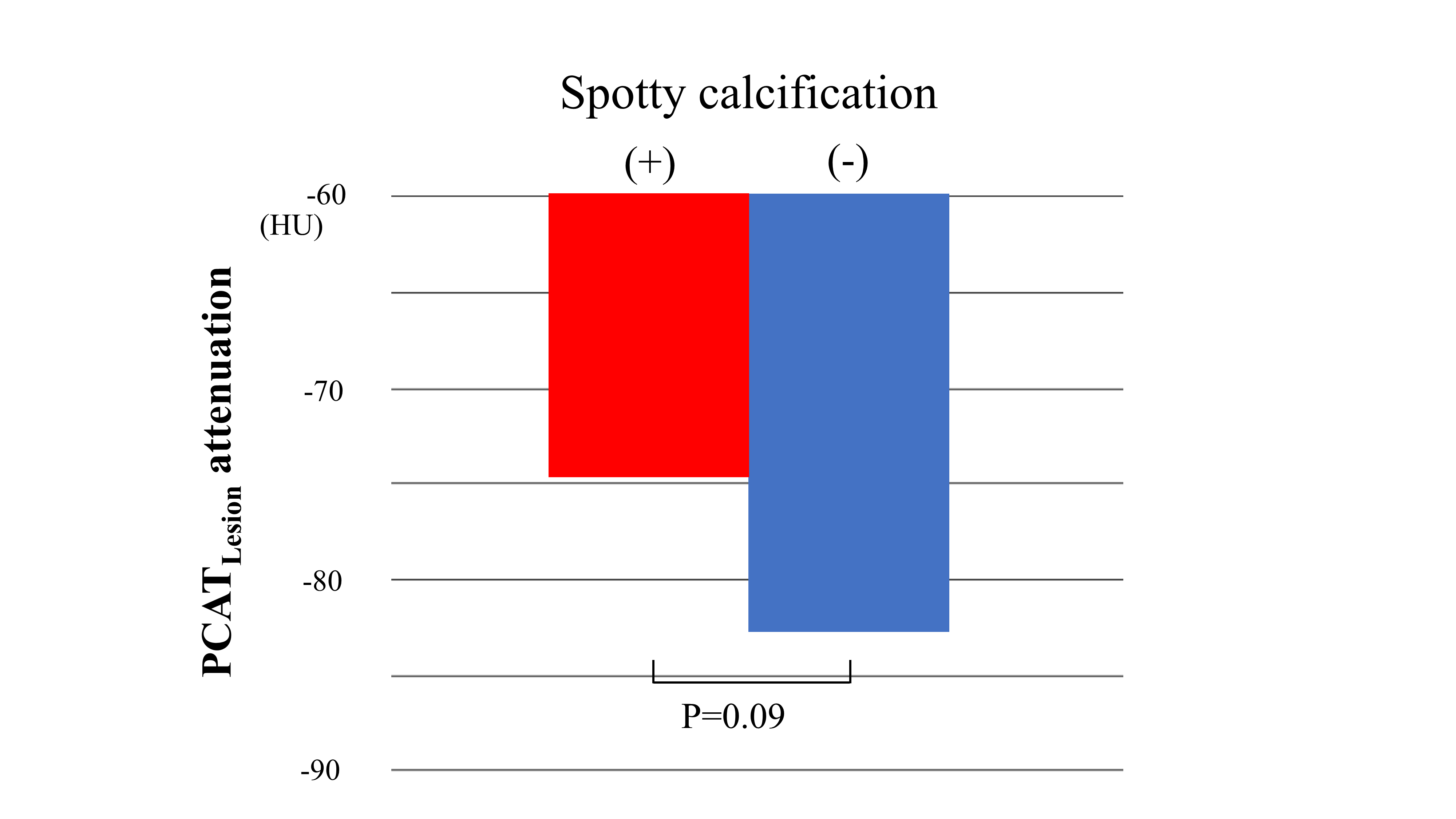

Supplement: Supplementary file 3 — Supplementary file3 (TIF 1887 kb)—The correlation between plaque features derived from NIRS/IVUS and PCATLesion attenuation. a MaxLCBI4mm, b Plaque burden, c Remodeling index. IVUS= intravascular ultrasound, NIRS= near infrared spectroscopy, PCAT= pericoronary adipose tissue [file 10554_2023_2907_MOESM3_ESM.tif]

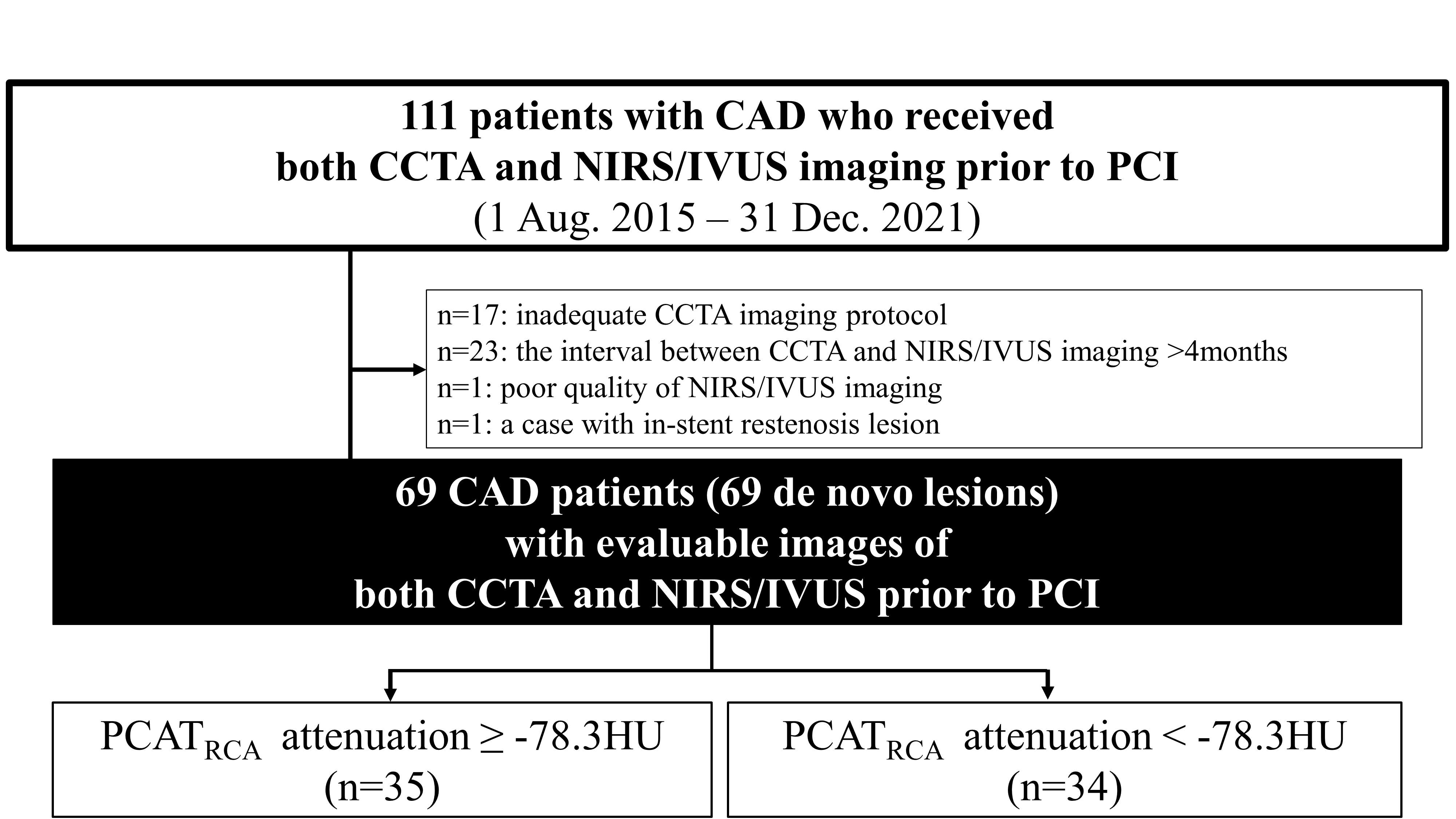

Supplement: Supplementary file 4 — Supplementary file4 (TIF 487 kb)—The relationships of PCATLesion attenuation with spotty calcification. PCAT= pericoronary adipose tissue [file 10554_2023_2907_MOESM4_ESM.tif]
